# Galectin-1 inhibition attenuates profibrotic signaling in hypoxia-induced pulmonary fibrosis

**DOI:** 10.1038/cddiscovery.2017.10

**Published:** 2017-04-10

**Authors:** Jaymin J Kathiriya, Niyati Nakra, Jenna Nixon, Puja S Patel, Vijay Vaghasiya, Ahmed Alhassani, Zhi Tian, Diane Allen-Gipson, Vrushank Davé

**Affiliations:** 1 Department of Pathology and Cell Biology, Morsani College of Medicine, University of South Florida, Tampa, FL 33612, USA; 2 University of Miami, Coral Gables, FL 33124, USA; 3 Department of Pharmaceutical Science, College of Pharmacy, University of South Florida, Tampa, FL 33612, USA; 4 Department of Cancer Biology and Evolution, H Lee Moffitt Cancer Center and Research Institute, Tampa, FL 33612, USA

## Abstract

Idiopathic pulmonary fibrosis (IPF) is characterized by lung remodeling arising from epithelial injury, aberrant fibroblast growth, and excessive deposition of extracellular matrix. Repeated epithelial injury elicits abnormal wound repair and lung remodeling, often associated with alveolar collapse and edema, leading to focal hypoxia. Here, we demonstrate that hypoxia is a physiological insult that contributes to pulmonary fibrosis (PF) and define its molecular roles in profibrotic activation of lung epithelial cells. Hypoxia increased transcription of profibrotic genes and altered the proteomic signatures of lung epithelial cells. Network analysis of the hypoxic epithelial proteome revealed a crosstalk between transforming growth factor-*β*1 and FAK1 (focal adhesion kinase-1) signaling, which regulated transcription of galectin-1, a profibrotic molecule. Galectin-1 physically interacted with and activated FAK1 in lung epithelial cells. We developed a novel model of exacerbated PF wherein hypoxia, as a secondary insult, caused PF in mice injured with subclinical levels of bleomycin. Hypoxia elevated expression of phosphorylated FAK1, galectin-1, and *α*-smooth muscle actin and reduced caspase-3 activation, suggesting aberrant injury repair. Galectin-1 inhibition caused apoptosis in the lung parenchyma and reduced FAK1 activation, preventing the development of hypoxia-induced PF. Galectin-1 inhibition also attenuated fibrosis-associated lung function decline. Further, galectin-1 transcript levels were increased in the lungs of IPF patients. In summary, we have identified a profibrotic role of galectin-1 in hypoxia signaling driving PF.

## Introduction

Idiopathic pulmonary fibrosis (IPF), a disease of unknown etiology, causes over 80 000 deaths per year in the United States and Europe combined.^1^ Despite this health burden, molecular basis of IPF remains poorly understood, leading to inadequate treatment options. While persistent accumulation of extracellular matrix (ECM) proteins and differentiation of lung fibroblasts to collagen-secreting myofibroblasts is a hallmark of IPF, the role of dysfunctional lung epithelium in IPF has only recently emerged.^2,3^ Chronic repetitive injuries to the lung epithelium leads to aberrant epithelial repair associated with pathogenic cellular reprograming, causing IPF.^4^ Chronic hypoxia of the lung is one such injury and a significant clinical feature of patients with IPF.^2^ This is particularly true in the aged with a history of lung infections and other interstitial lung diseases (ILDs). Chronic ILDs cause progressive decline in lung function associated with reduced elastic recoil, chest wall compliance, and loss of intercostal muscle mass, leading to restrictive ventilatory response and hypoxemia.^5–7^ Indeed, worsening levels of hypoxemia, which may occur because of impaired alveolar gas exchange, is a clinical feature of IPF patients undergoing acute exacerbations of IPF (AE-IPF).^2,3,7–9^

The lungs of IPF patients are often characterized by the presence of fibroblastic foci surrounded by hyperplastic type II alveolar epithelial cells (AEC2s),^10^ which become hypoxic and express increased levels of hypoxia-inducible factor-1*α* (HIF-1*α*).^11^ Further, large-scale genomic studies on lungs of IPF patients indicate that hypoxia signaling likely drives profibrotic events in IPF.^12^ Sustained hypoxia signaling in the AEC2s can contribute to increased production of hypoxia-inducible profibrotic genes such as transforming growth factor-*β*1 (TGF-*β*1).^13–15^ Since wound repair-associated processes are highly regulated by the presence of oxygen,^16^ hypoxic microenvironment due to compromised microcirculation can cause deregulation of wound repair in AEC2s. Indeed, hypoxia can induce profibrotic activities via aberrant regulation of cytoskeletal remodeling in AEC2s.^17^ Therefore, it is important to define the role of hypoxia signaling and their effectors in AEC2s in the pathogenesis of IPF.

Profibrotic signaling pathways such as Wnt/*β*-catenin and TGF-*β* influence cytoskeletal remodeling and increase cell plasticity in AEC2s, contributing to aberrant epithelial repair in fibrotic lungs.^18,19^ Many of these activities are mediated by FAK1 (focal adhesion kinase-1), a non-receptor tyrosine kinase and a master regulator of cytoskeletal remodeling.^20,21^ FAK1 has critical roles in fibroblast to myofibroblast differentiation.^22^ Although elevated expression of activated/phosphorylated FAK1 (pFAK1) is often observed in the lungs of IPF patients,^23^ the molecular mechanisms of FAK1 activation in pulmonary hypoxia remain unknown.

In this study, we define the role of hypoxia in profibrotic activation of lung epithelial cells via FAK1 and galectin-1. Our studies revealed that galectin-1 is a novel regulator of FAK1 in hypoxic lung epithelial cells. Galectin-1 is a hypoxia-responsive protein that contributes to invasion, migration, and survival of lung cancer cells.^24,25^ We observed that galectin-1 interacted with and increased FAK1 phosphorylation in lung epithelial cells. Our mouse model suggests that hypoxia can contribute to increased fibrosis in the lung via galectin-1 and reduced apoptosis in the lung parenchyma. Galectin-1 inhibition increased apoptosis in the fibrotic lungs and attenuated lung function decline associated with hypoxia-induced pulmonary fibrosis (PF). Further, galectin-1 transcripts were increased in hyperplastic regions of the lungs of IPF patients, suggesting that galectin-1 may contribute to hyperplasia of the lung epithelium. In summary, our studies highlight galectin-1 as a novel hypoxia-responsive profibrotic molecule in epithelial cells, which is amenable to therapeutic targeting in PF.

## Results

### Hypoxia increased cell plasticity, proliferation, and migration of lung epithelial cells

**Figure 1:**
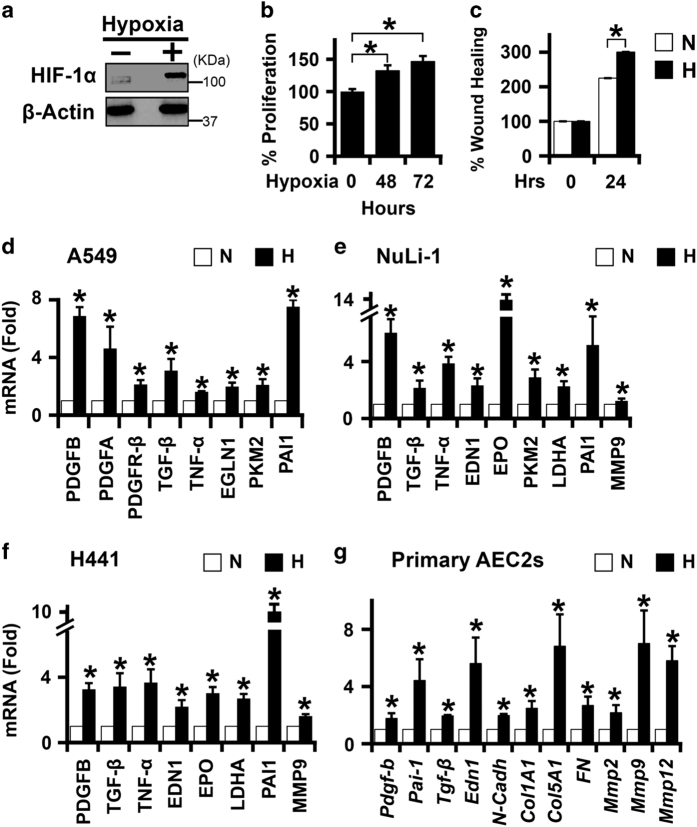
Hypoxia reprogramed and increased cell plasticity, proliferation, and migration of H441 cells. (**a**) Stabilization of HIF-1*α* protein expression under hypoxia (1% O_2_; 24 h) as compared with normoxia (21% O_2_; 24 h) in H441 lung epithelial cells. (**b**) Increased cell proliferation of H441 cells exposed to 48 and 72 h of hypoxia (1% O_2_) as compared with normoxic control cells (0 h). First lane (72 h normoxia; 0 h hypoxia); second lane (24 h normoxia followed by 48 h hypoxia); third lane (0 h normoxia; 72 h hypoxia); (*n*=8 in each experimental group). *P*<0.05 (Student’s *t*-test). Data are shown as mean±S.E. (**c**) Increased rate of wound closure in H441 cells under hypoxia (1% O_2_) as compared with normoxic control cells (solid bars: hypoxia, H; open bars, normoxia, N). Total of three scratches were made in each condition (three wells per condition). Ten images at ×40 were taken of each scratch for quantitation. *P*<0.05 (Student’s *t*-test). Data are shown as mean±S.E. (**d**–**g**) Increased expression of mRNAs of profibrotic genes in epithelial cells under hypoxia. A549, H441, and NuLi-1 cells were exposed to 1% O_2_ for 24 h. Primary murine AEC2s were exposed to 1% O_2_ for 48 h. *P<0.05 (Student’s *t*-test). Data are shown as mean±S.E. (*n*=3 independent experiments).

Exposure to hypoxia increased proliferation and migration of H441 lung epithelial cells (Figures 1a–c). To determine the effect of hypoxia as a profibrotic injury to distinct lung epithelial cells, four different cell types derived from proximal and distal lung epithelium were exposed to hypoxia. These include NuLi-1 cells: primary bronchial epithelial cells; H441 cells: derived from bronchoalveolar acinar region, which maintains alveolar and club cell-like features that exhibit features of AEC2s;^26^ A549 cells: originating from AEC2s; and primary murine AEC2s. Hypoxia increased mRNA levels of a host of profibrotic genes, including *PDGFB* (platelet-derived growth factor B), *TGF-β*, *TNF-α* (tumor necrosis factor-*α*), *EDN1* (endothelin-1), and *PAI-1* (plasminogen activator inhibitor-1) (Figures 1d–g) in each cell type. Similarly, hypoxia increased mRNA levels of ECM proteins (collagens, fibronectin, and matrix metalloproteases) in isolated primary murine AEC2s (Figure 1g and Supplementary Figure S1), consistent with the observation of epithelial plasticity in lungs of IPF patients.^27^ In summary, our findings indicate that epithelial hypoxia is a profibrotic insult capable of altering the lung matrix as seen in IPF.

### Stable isotope labeling by amino acids in cell culture-based LC-MS/MS proteomics identified TGF-*β* and Wnt3a as regulators of hypoxic proteome

**Figure 2:**
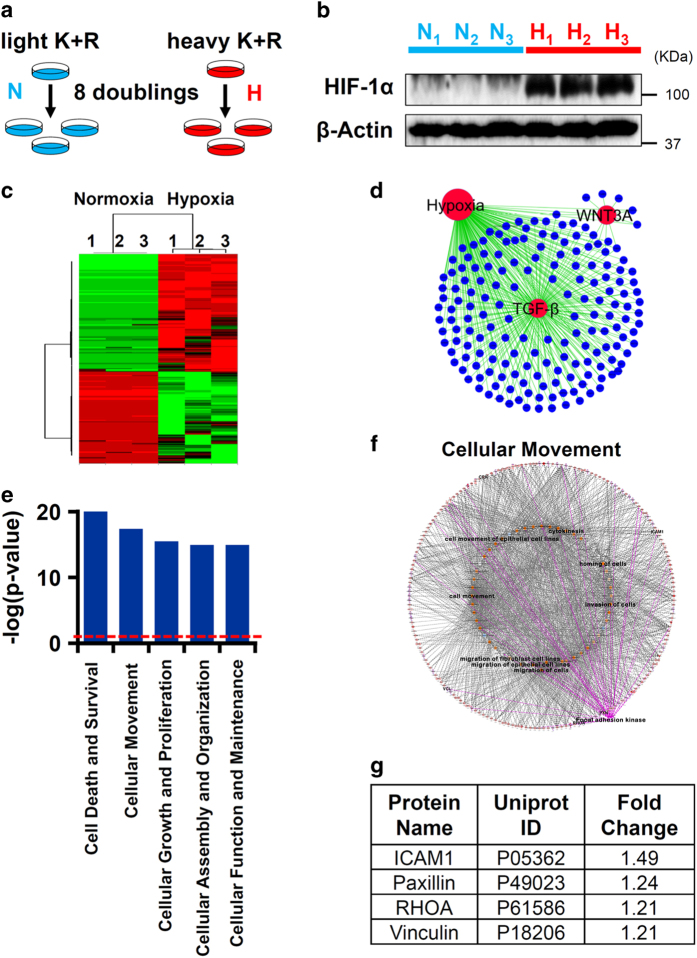
Stable isotope labeling by amino acids in cell culture (SILAC)-based liquid chromatography tandem-mass spectrometry (LC-MS/MS) proteomics identified TGF-*β* and Wnt3a as regulators of hypoxic proteome. (**a**) H441 cells were grown in culture media containing either light lysine/arginine (blue) or heavy lysine (U-^13^C_6_)/arginine (U-^13^C_6_,^15^N_6_; red) for eight cell doublings to achieve complete incorporation of isotopes. Cells labeled with heavy lysine/arginine were exposed to hypoxia (1% O_2_; 72 h) and light lysine/arginine-labeled cells were grown in normoxic conditions (21% O_2_). (**b**) Protein samples subjected to SILAC-proteomics had stabilized HIF-1*α*, indicating that sustained hypoxia signaling was achieved. (**c**) Heat map of 1476 proteins with significantly altered expression levels in at least two samples (Mann–Whitney test, *P*<0.05) was generated using the XLSTAT software (New York, NY, USA). Red: increased expression and green: decreased expression of proteins following hypoxia treatment. (**d**) Upstream network analysis using IPA identified TGF-*β* and Wnt3a as significantly upregulated upstream regulators of hypoxia-deregulated proteins. Upstream regulators hypoxia, TGF-*β*, and Wnt3a are depicted as red circles. Hypoxia-deregulated proteins, which are influenced by TGF-*β* and Wnt3a, are depicted as blue circles. (**e**) Functional enrichment of the 1476 proteins was performed using IPA. Top five altered cellular and molecular processes are depicted. The red line indicates *P*=0.05 (right-tailed Fisher’s exact test). (**f**, **g**) Functional crosstalk between cellular migration-related proteins identified in our proteomics revealed FAK1 as a regulator of cellular migration. Red color in the network depicts upregulated proteins in hypoxic proteome. Pink color depicts downregulated proteins. Significantly enriched functions of the proteins involved in cellular migration network are labeled. FAK1-effector proteins are highlighted and listed.

Proteomics analysis of normoxic and hypoxic epithelial cells identified 1476 significantly deregulated proteins (Figures 2a–c). Upstream analysis of these proteins identified targets of TGF-*β* and Wnt3a to be significantly enriched in the hypoxic proteome (Figure 2d). Bioinformatics-based functional enrichment of the hypoxic proteome identified important cellular processes such as cell death, cellular movement, cellular growth and proliferation, and cellular assembly and organization (Figure 2e). As these processes are initiated during cytoskeletal remodeling in aberrantly activated lung epithelial cells of IPF patients,^4^ we sought to identify molecules causing epithelial abnormalities. Functional network analysis on our proteomics data (Figure 2f) revealed that cytoskeletal proteins central to FAK1 functions such as RHOA, paxillin, ICAM1, and vinculin^21,28^ were increased in hypoxic epithelial cells (Figure 2g). In summary, our proteomics analysis suggested that hypoxia activates FAK1 signaling and contributes to activation of cytoskeletal remodeling in lung epithelial cells.

### FAK1 is activated by hypoxia and regulated TGF-*β* signaling in lung epithelial cells

**Figure 3:**
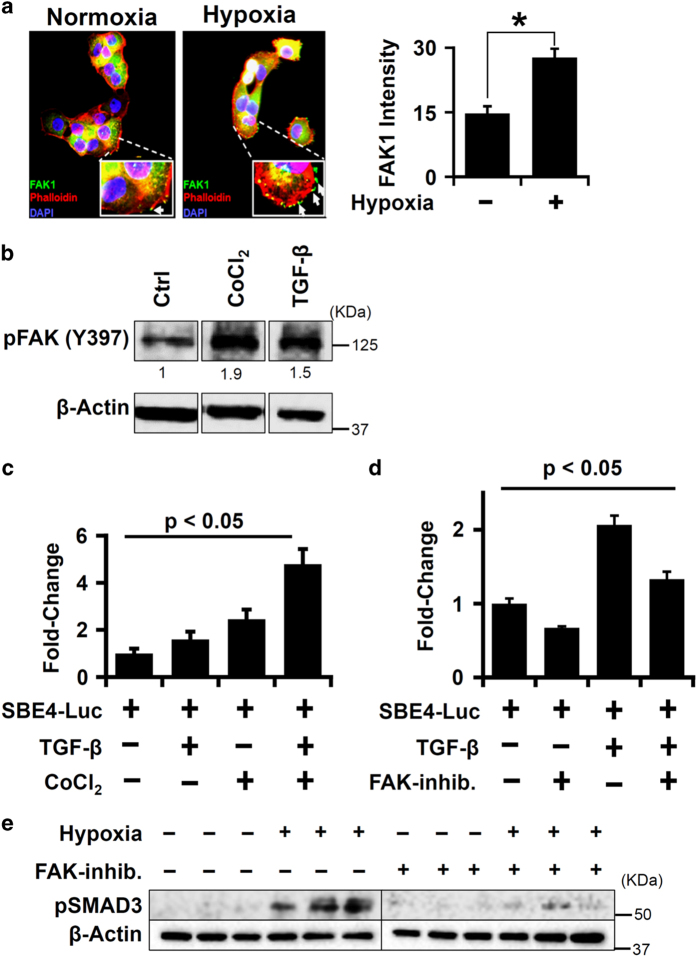
FAK1 is activated by hypoxia and regulated TGF-*β* signaling in lung epithelial cells. (**a**) Membrane localization of FAK1 was increased twofold under hypoxia in H441 cells (1% O_2_; 6 h). FAK1 intensity was calculated by analyzing a total of 10 fields at ×10. Experiment was repeated three times. *P<0.05 is assumed as significant (Student’s *t*-test). Data are shown as mean±S.E. (**b**) Significantly increased pFAK1 (Y397) levels in H441 cells after exposure to hypoxia mimic, CoCl_2_ (100 *μ*M, 15 min) or TGF-*β* (5 ng/ml; 15 min). *P*<0.05 (Student’s *t*-test). Data are shown as mean (*n*=3 independent experiments). (**c**) Increased SMAD-mediated transcriptional activity following hypoxia and TGF-*β* treatments. H441 cells were transfected with SMAD reporter plasmid (SBE4-Luc; 2 *μ*g per well) and treated with either TGF-*β* (5 ng/ml) or CoCl_2_ (100 *μ*M) or both. *P*<0.05 (Student’s *t*-test). Data are shown as mean±S.E. (*n*=3 independent experiments). (**d**) Inhibition of FAK1 reduced TGF-*β*-independent and -dependent SMAD-mediated transcriptional activity. H441 cells were transfected with SBE4-Luc plasmid and treated with TGF-*β* (5 ng/ml) or FAK inhibitor PF-573 228 (10 *μ*M) or both. *P*<0.05 (Student’s *t*-test). Data are shown as mean±S.E. (*n*=3 independent experiments). All transfection experiments (c and d) were performed at least two times in triplicates and normalized against *β*-galactosidase activity. (**e**) FAK1 inhibition reduced hypoxia-induced pSMAD3 levels. H441 cells were treated with or without FAK inhibitor PF-573  228 (10 *μ*M) for 1 h before treating with hypoxia (1% O_2_; 12 h).

We examined whether hypoxia activated FAK1 as it has important roles in cell migration^21^ and was identified as a central molecule in our network analysis and. Hypoxia increased membrane localization of FAK1 by twofold in H441 cells (Figure 3a). We also observed that TGF-*β*, Wnt3a, and hypoxia treatment significantly increased pFAK1 (Y397) levels (Figure 3b and Supplementary Figure S2a), indicating activation of FAK1. As our bioinformatics analysis enriched TGF-*β* and Wnt3a proteins as regulators of the hypoxic proteome (Figure 2), we wanted to ascertain whether TGF-*β* and Wnt signaling pathways engage in intracellular crosstalk in hypoxic lung epithelial cells. Indeed, TGF-*β* and hypoxia signaling increased *β*-catenin transcriptional activity (Supplementary Figure S2b), suggesting a functional TGF-*β*/Wnt crosstalk that can activate lung epithelial cells. Hypoxia also increased endogenous SMAD activity, which was further induced by TGF-*β* as assessed by SMAD-induced promoter-reporter activity in H441 cells (Figure 3c). Further, we tested whether FAK1 had a role in hypoxia-dependent TGF-*β*/SMAD signaling pathway. Indeed, FAK1 inhibition attenuated SMAD-mediated transcriptional activity as well as hypoxia-dependent SMAD3 phosphorylation in lung epithelial cells (Figures 3d and e). In summary, our results implicate critical roles of FAK1 in hypoxia-mediated TGF-*β*/SMAD signaling pathway in lung epithelial cells.

### FAK1 knockdown dampened profibrotic activities of lung epithelial cells

**Figure 4:**
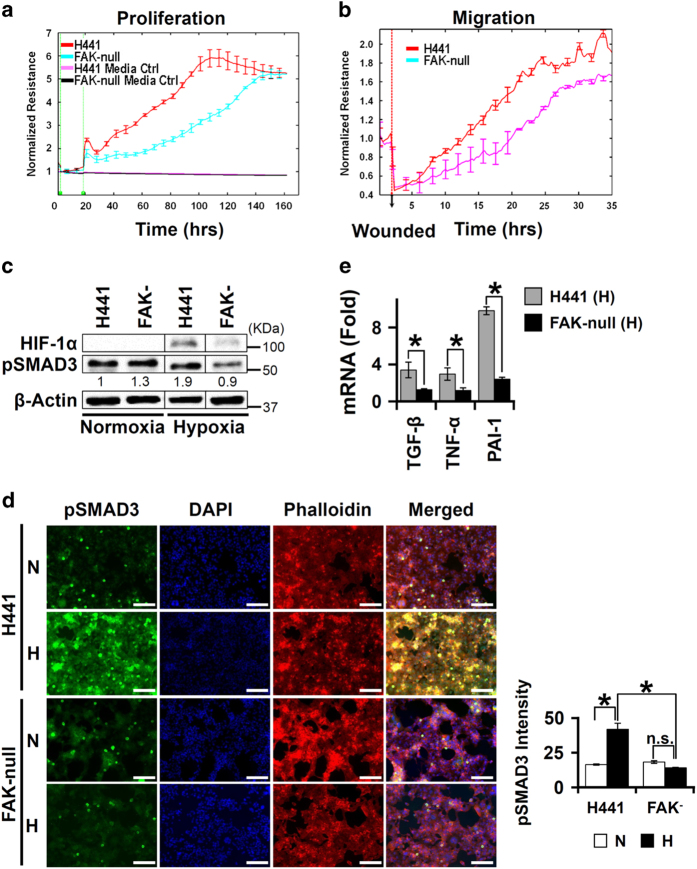
FAK1 knockdown dampened profibrotic activities of lung epithelial cells (**a**, **b**) Compromised proliferative and migratory capabilities of FAK-null H441 cells as compared with normal H441 cells as determined by ECIS. *P*<0.05 (Student’s *t*-test). Data are shown as mean±S.E. (*n*=4 independent experiments). (**c**) FAK1 knockdown significantly dampened hypoxia-induced pSMAD3 levels when compared with normal H441 cells as determined via immunoblotting. *P*<0.05 (Student’s *t*-test). Data are shown as mean (*n*=3 independent experiments). (**d**) Reduced pSMAD3 levels due to FAK1 knockdown were confirmed via ICC analysis. Normal and FAK-null H441 cells were plated onto coverslips and allowed to reach 100% confluence. Cells were serum starved for 24 h before hypoxia treatment (H; 12 h; 1% O_2_) or normoxia treatment (N; 12 h; 21% O_2_). Quantitation of pSMAD3 intensity was performed on images of at least three fields at ×10 magnification in each conditions. Experiment was repeated three times. *P<0.05 (Student’s *t*-test). NS=not significant. Data are shown as mean±S.E. (*n*=3 independent experiments). (**e**) Loss of FAK1 abrogated hypoxia-mediated induction of profibrotic mediators TGF-*β*, TNF-*α*, and PAI-1 compared with normal H441 cells as determined by qRT-PCR. *P<0.05 (Student’s *t*-test). Data are shown as mean±S.E. (*n*=3 independent experiments). NS, not significant.

Our bioinformatics analysis of the hypoxic proteome suggested that FAK1 may have a central role in profibrotic activities of lung epithelial cells. Indeed, FAK1 knockdown in H441 cells (FAK-null; Supplementary Figure S3) inhibited proliferative and wound healing capabilities as compared with parent H441 cells (Figures 4a and b). Consistent with our earlier experiment where FAK1 inhibition reduced hypoxia-mediated SMAD3 activation (Figure 3e), stable knockdown of FAK1 reduced hypoxia-induced SMAD3 phosphorylation (Figures 4c and d). To determine whether FAK1 knockdown also affected SMAD3-dependent gene transcription in hypoxia, we tested mRNA levels of profibrotic genes that are hypoxia- and SMAD-responsive. We observed that FAK1 deficiency attenuated hypoxia-induced expression of TGF-*β*, TNF-*α*, and PAI-1 mRNAs (Figure 4e). Thus, FAK1 modulates profibrotic activities in normal lung epithelial cells by controlling their proliferation and migration and by regulating hypoxia-mediated TGF-*β*/SMAD signaling pathway.

### Galectin-1 and FAK1 mutually activated each other in hypoxic lung epithelial cells

**Figure 5:**
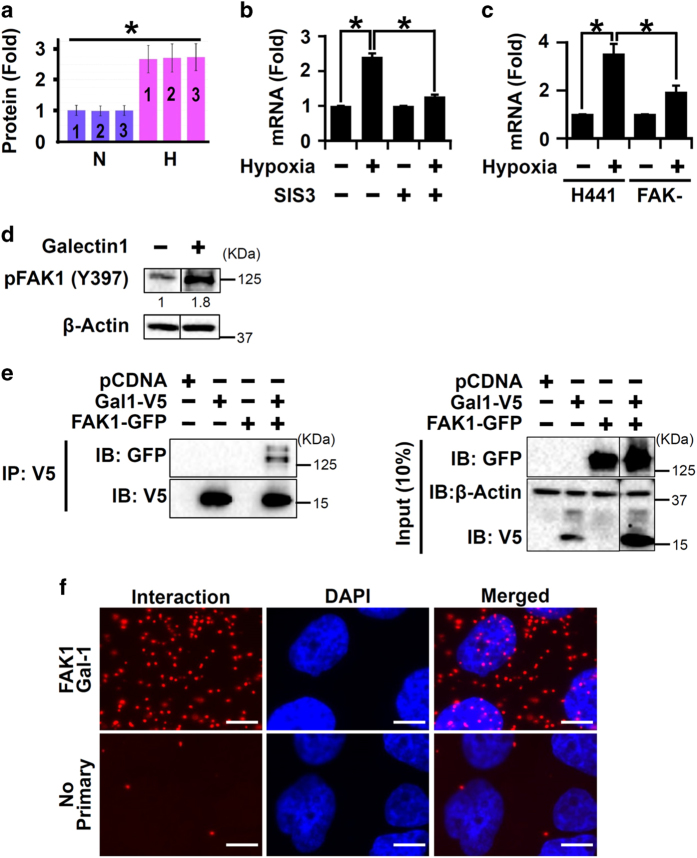
Galectin-1 and FAK1 mutually activated each other in hypoxic lung epithelial cells. (**a**) Galectin-1 protein levels were increased following hypoxia treatment (1% O_2_; 72 h) as determined by LC-MS/MS in H441 cells. *P<0.05 (Mann–Whitney *U*-test). Data are shown as mean±S.D. (*n*=3 independent experiments). (**b**) Loss of FAK1 H441 reduced galectin-1 mRNA expression in hypoxic H441 cells. FAK-null (FAK−) H441 cells were exposed to hypoxia for 24 h followed by quantitation of mRNA levels of galectin-1 via qRT-PCR. *P<0.05 (Student’s *t*-test). Data are shown as mean±S.E. (*n*=3 independent experiments). (**c**) SMAD3 inhibition reduced galectin-1 mRNA expression in hypoxic H441 cells. Cells were pretreated with SMAD3 inhibitor SIS3 (3 *μ*M; 2 h) or vehicle followed by exposure to hypoxia (1% O_2_; 24 h) and mRNA levels of galectin-1 were determined by qRT-PCR. *P<0.05 (Student’s *t*-test). Data are shown as mean±S.E. (*n*=3 independent experiments). (**d**) Galectin-1 treatment increased pFAK (Y397) levels in H441 cells. H441 cells were serum starved for 24 h and then treated with recombinant galectin-1 (50 ng/ml; 15 min). *P*<0.05 (Student’s *t*-test). Data are shown as mean (*n*=3 independent experiments). (**e**) Co-IP experiment detected interaction between galectin-1 and FAK1. HEK-293T cells were transfected with plasmid vectors expressing either V5-galectin-1 (lane 2) or GFP-FAK1 (lane 3) or both (lane 4). Cells transfected with empty vector (pCDNA, lane 1) were used as control. IP with V5 antibody and immunoblotting (IB) with GFP antibody revealed interaction between galectin-1 and FAK1 (lane 4). (**f**) PLA performed in H441 cells demonstrated physical interaction of endogenous galectin-1 and FAK1 proteins. Each red dot indicates interaction between the two proteins (scale bar=10 *μ*m). No primary was used as a negative control, which displays minimal background staining. Images are representative of two independent experiments.

As our systems biology approach identified a central role of FAK1 in profibrotic activation of lung epithelial cells, we analyzed the proteome derived from hypoxic epithelial cells to identify novel regulators of FAK1. Galectin-1, a known hypoxia-responsive protein,^24^ is known to increase the migratory potential of lung cancer cells.^29^ Further, galectin-1 expression is induced by TGF-*β*/SMAD signaling in lung fibroblasts, accelerating PF.^30^ As FAK1 knockdown dampened SMAD3 activation in hypoxia (Figures 4c and d), we tested whether SMAD3 and FAK1 can regulate hypoxia-induced transcription of galectin-1. Indeed, FAK1 knockdown and SMAD3 inhibition reduced hypoxia-induced galectin-1 mRNA levels (Figures 5b and c), indicating a functional crosstalk of SMAD3 and FAK1 signaling to regulate galectin-1 activation in hypoxic lung epithelial cells. As galectin-1 is associated with increased cellular proliferation and migration,^29^ we tested for a functional relationship between FAK1 and galectin-1. Treatment with galectin-1 increased phosphorylation of FAK1 (~1.8-fold), whereas inhibition of galectin-1 by OTX008^31^ reduced hypoxia-induced pFAK1 levels in H441 cells (Figure 5d and Supplementary Figure S4b). Thus, galectin-1 contributes to hypoxia-mediated FAK1 activation. Whether galectin-1 physically interacts with FAK1 to influence its function is not known. Our co-immunoprecipitation (co-IP) experiment revealed that FAK1 and galectin-1 interacted in HEK-293T cells (Figure 5f). Further, physical interaction between endogenous FAK1 and galectin-1 in H441 cells was detected by PLA (proximity ligation assay; Figure 5g). In summary, galectin-1 activates and interacts with FAK1, likely contributing to hypoxia-mediated activation of FAK1 in lung epithelial cells.

### Hypoxia caused exacerbation of fibrosis via galectin-1 in mice injured with low levels of bleomycin

**Figure 6:**
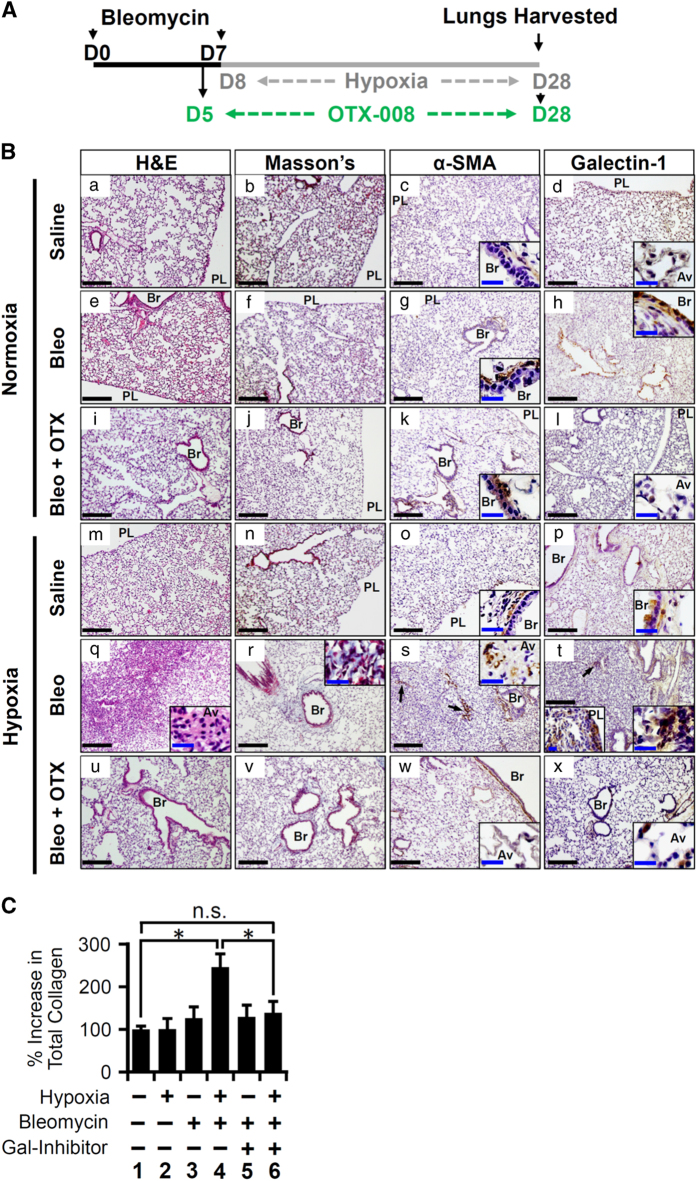
Hypoxia causes exacerbation of fibrosis via galectin-1 in mice injured with low levels of bleomycin. (**A**) Experimental design depicting two bleomycin insults (day 0—D0; day 7—D7) followed by hypoxia treatment for 3 weeks (day 8 to day 28). Galectin-1 inhibitor (OTX008) treatment was performed from day 5 to day 28. (**B**) Hypoxia treatment of mice injured with bleomycin induced fibrosis. Mice were exposed to hypoxia alone (m–p) or treated with intratracheal bleomycin alone (e–h) or underwent hypoxia and bleomycin insults together (q–t). Mice were also treated with galectin-1 inhibitor (i–l, u–x) or saline (e–h, q–t). H&E staining (a, e, i, m, q, u), Masson’s trichrome staining for visualization of collagen deposition (b, f, j, n, r, v), *α*-SMA staining (c, g, k, o, s, w), and galectin-1 staining (d, h, l, p, t, x) was performed. Black scale bars=100 *μ*m; blue scale bars=20 *μ*m. *α*-SMA expression is increased in the proximal parenchyma (s; arrows) and distal parenchyma (s, inset) of mouse lungs treated with hypoxia and bleomycin. Similarly, galectin-1 expression is increased in proximal parenchyma (t; arrow and right bottom inset) and distal parenchyma (left bottom inset). Galectin-1 inhibition reversed lung remodeling (u), collagen deposition (v), and reduced *α*-SMA (w) and galectin-1 expression (x). (**C**) Galectin-1 inhibition reduced total collagen deposition in the lungs of mice injured with hypoxia and bleomycin. Total collagen was measured using sircol assay. *P<0.05 (one-way analysis if variance (ANOVA) was performed with *post hoc* one-tailed *t*-test). Data are shown as mean±S.E. (*n*=4 in each group). Av, alveolar region; br, pronchiolar region; NS, not significant; PL, pleural space.

Hypoxia signaling is often observed in IPF patients^12^ and is a clinical feature of patients with AE-IPF.^8^ This led us to hypothesize that hypoxia signaling may contribute to fibrosis in the lungs that have already undergone sustained microinjuries. To test this hypothesis, we established a novel mouse model. Herein, the lungs were mildly injured with two low doses of bleomycin (0.1 U) and then exposed to hypoxia (13% O_2_) for 3 weeks (Figure 6A). Subclinical injuries with bleomycin alone did not cause any detectable histological changes in the lung (Figure 6Be). However, we observed increased galectin-1 expression (Figure 6Bh), which may prime the lungs to become susceptible to develop fibrosis in the event of a physiological challenge like hypoxia. When mice with bleomycin-injured lungs were exposed to hypoxia, they developed PF (Figure 6Bq) when compared with saline-treated mice (Figure 6Bm). Mice challenged with both bleomycin and hypoxia had significantly increased collagen deposition (Figures 6Br and C, lane 4) compared with the mice treated with bleomycin (Figures 6Bf and C, lane 3) or hypoxia alone (Figures 6Bn and C, lane 2). This fibrotic phenotype was associated with increased expression of *α*-smooth muscle actin (SMA), a marker of myofibroblasts in the parenchyma (Figure 6Bs arrows). Galectin-1 was increased in distal (Figure 6Bt, left inset) and proximal parenchyma (Figure 6Bt, arrow, right inset). Therefore, it is likely that galectin-1 as a profibrotic molecule contributes to hypoxia-mediated exacerbation of PF. Thus, when lungs undergo subclinical microinjuries (bleomycin in our case), they are susceptible to develop fibrosis. Supporting this notion, secondary insults such as hypoxia associated with induction of profibrotic molecules such as galectin-1 is a likely mechanism that can lead to PF in our two-hit (bleomycin/hypoxia) model.

### Galectin-1 inhibition reduced collagen deposition and fibrotic phenotype observed in the exacerbated PF model

As we observed increased galectin-1 expression in our mouse model, we hypothesized that galectin-1 inhibition can attenuate hypoxia-induced PF. We used a galectin-1-specific small-molecule inhibitor, OTX008,^31^ which is currently in phase I clinical trial for cancer patients (trial identifier: NCT01724320). In mice treated with bleomycin alone, we observed no detectable effect of galectin-1 inhibitor on lung architecture (Figure 6Bi) and collagen deposition (Figures 6Bj and C, lane 5). Galectin-1 inhibition reduced lung remodeling (hematoxylin and eosin (H&E) staining; Figure 6Bu) and collagen deposition (Figures 6Bv and C, lane 6)) in lungs undergoing fibrosis. Further, a decrease in *α*-SMA expression in the lungs undergoing fibrosis (Figure 6Bw) indicated that galectin-1 inhibition dampened the progression of fibrosis. Thus, galectin-1 inhibitor can become a prospective therapy, providing novel clinical rationale for IPF treatment.

### Galectin-1 inhibition rescued hypoxia-induced lung function decline in bleomycin-injured mouse lungs

**Figure 7:**
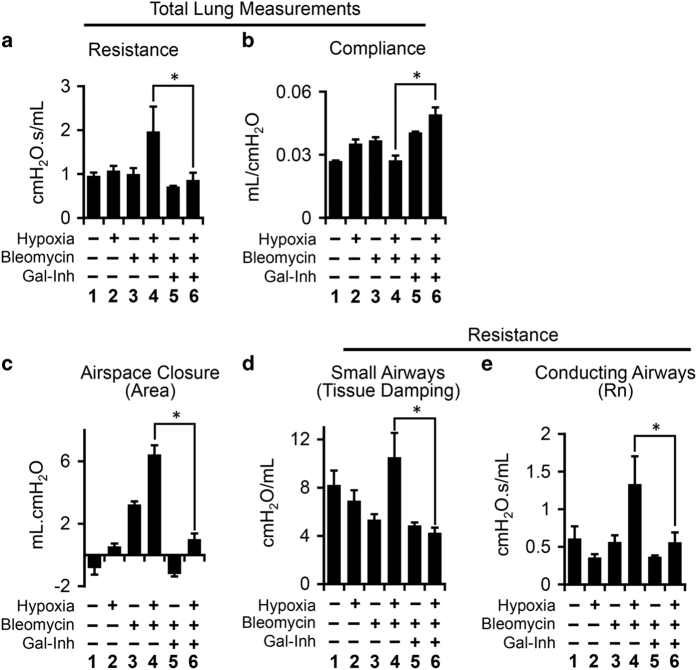
Galectin-1 rescues lung function decline caused by dual insults with hypoxia and bleomycin in mice. (**a**) Total lung resistance of mouse lungs was increased due to hypoxia treatment of mice injured with bleomycin, which was rescued by galectin-1 inhibition. Lung function tests were performed using the flexiVent system (SCIREQ Technologies Inc.). Lung resistance was measured using Snapshot-150 perturbation maneuver. (**b**) Total lung compliance of mouse lungs decreased because of hypoxia treatment of mice injured with bleomycin. Galectin-1 inhibitor treatment significantly increased lung compliance. Lung compliance was measured using Snapshot-150 perturbation maneuver. (**c**) Airspace closure, reflective of airway collapse, increased in mice treated with hypoxia and bleomycin. The airway collapse was reduced by galectin-1 inhibitor treatment. Airspace closure was measured using pressure–volume loop (PVs-P) perturbation. (**d**) Resistance of small airways (terminal bronchioles and alveoli) increased because of hypoxia and bleomycin injuries. Measured using QuickPrime-3 perturbation maneuver. (**e**) Conducting airways (airways larger than 500 *μ*m in diameter in mouse lungs) increased due to hypoxia and bleomycin treatment. Galectin-1 inhibition significantly reduced the increase airway resistance. Measured using QuickPrime-3 perturbation maneuver. All experiments were performed in at least four mice in every group. One-way analysis variance (ANOVA) was performed with *post hoc* one-tailed *t*-test to measure significance at *P*<0.05 (*). Data are shown as mean±S.E.

IPF patients suffer from gradual decline in lung function, which worsens during phases of AE associated with hypoxemia.^8,32^ Based on above results, we hypothesized that hypoxia could be a major contributor in lung function decline, which can be rescued by galectin-1 inhibition. To test this hypothesis, we performed lung function tests on all groups of mice on day 28 after initial bleomycin insult followed by hypoxia (Figure 7a). Total lung resistance was increased in the lungs of mice challenged with bleomycin and exposed to hypoxia, which was reduced by galectin-1 inhibition (Figure 7a, lanes 4 and 6). Similarly, total lung compliance decreased owing to bleomycin and hypoxia challenge, while galectin-1 inhibition significantly increased total lung compliance (Figure 7b, lanes 4 and 6). Since IPF is characterized by lung remodeling, which is often associated with alveolar collapse,^2^ we measured the area covered by airspace closure, indicative of collapsed airways.^33^ Mice with PF had a sevenfold increase in airspace closure (Figure 7c, lane 4), which was significantly reduced by galectin-1 inhibitor (Figure 7c, lane 6). Similarly, hypoxic lung injury to the mice challenged with bleomycin significantly increased resistance of both small airways and conducting airways, as assessed by tissue damping and airway resistance, respectively (Figures 7d and e, lane 4). Galectin-1 inhibition rescued hypoxia-induced resistance in both conducting and small airways (Figures 7d and e, lane 6). In summary, Galectin-1 inhibitor has the potential to inhibit PF associated with declining lung function in IPF patients.

### Galectin-1 inhibition attenuates FAK1 activation and induces apoptosis in hypoxia- and bleomycin-injured mouse lungs

Hyperplastic epithelial cells in the lungs of IPF patients are characterized by activated hypoxia signaling.^11^ Therefore, it is possible that galectin-1, which is regulated by hypoxia and FAK1, can also activate FAK1 and increase epithelial proliferation in hypoxic lungs. Indeed, our data suggest that pFAK1 (Y397) levels were significantly increased in the lungs of mice challenged with bleomycin and exposed to hypoxia, while galectin-1 inhibition significantly reduced pFAK1 levels (Supplementary Figure S4a). Further, galectin-1 inhibition activated caspase-3 in the lungs of mice injured with bleomycin and hypoxia (Supplementary Figure S4a), indicating that galectin-1 regulates hypoxia-mediated profibrotic effects in the lung. Our data suggest that galectin-1 inhibition can induce apoptotic pathway in hyperactivated epithelial cells via reduced FAK1 activation, which could potentially contribute to resolution of PF.

### Galectin-1 transcript levels are increased in IPF patients

**Figure 8:**
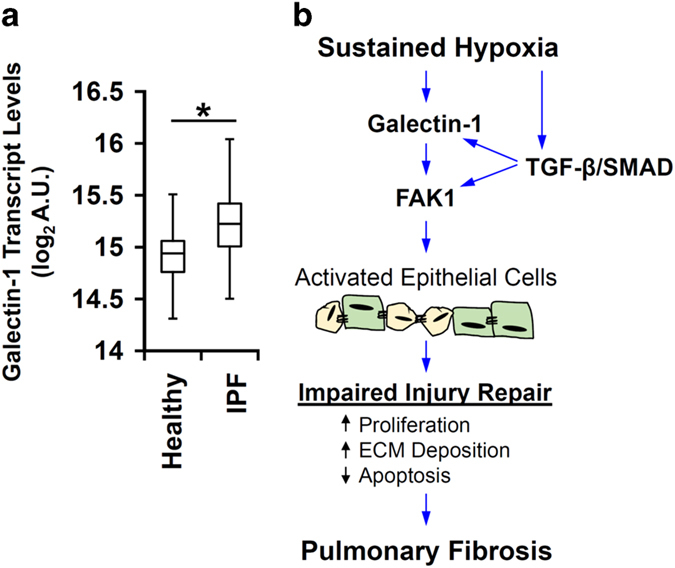
Hypoxia activates galectin-1 to drive aberrant epithelial activation, causing PF. (**a**) Log-transformed values of galectin-1 transcript are depicted using a box-whisker plot in arbitrary units (AU). Publically available data set of microarray profile of a total of 108 healthy and 160 patients with IPF was used to determine galectin-1 transcript levels (GSE47460). *P<0.0001 (Mann–Whitney *U*-test between two variables). (**b**) Hypoxia causes fibrosis by activating lung epithelial cells via galectin-1 and FAK1.

We analyzed the microarray data set from the lung samples of 160 IPF patients and 108 healthy controls available from lung tissue research consortium (Supplementary Tables S1 and S2; GEO accession ID: GSE47460). Galectin-1 levels are indeed upregulated in the lungs of IPF patients when compared with the lungs of healthy controls (Figure 8a). Since our study has identified a profibrotic role of galectin-1 in lung epithelial cells, we wanted to test whether this was indeed true for epithelium in the lungs of IPF patients. We analyzed a different microarray data set derived from different regions of the lungs of IPF patients (GEO accession ID: GSE35309). Galectin-1 transcripts were increased specifically in the hyperplastic regions in the lungs of IPF patients (Supplementary Figure S5). As activated hypoxia signaling is associated with IPF pathogenesis and hyperplastic AEC2s,^12^ it is plausible that hypoxia may have an important role in increasing galectin-1 levels in the hyperplastic regions in the lungs of IPF patients.

## Discussion

Lungs of IPF patients are characterized by dysfunctional epithelial cells and excessive ECM deposition causing aberrant lung remodeling associated with alveolar collapse.^3^ Alveolar edema and collapse may compromise microcirculation adjacent to the AECs eliciting focal hypoxia in the distal lung.^2^ Local hypoxia worsens the lung injury as oxygenated blood supply is diverted due to a remodeled endothelium away from permanently collapsed airways.^34^ Particularly, when this happens in an IPF patient, it may contribute to AE-IPF, which is characterized by elevated hypoxemia.^3,35–37^ However, the etiology of AE-IPF remains elusive. Herein, using multiple approaches, we have identified galectin-1 and FAK1 as contributors of epithelial hypoxic injury, likely causing progression and exacerbation of PF.

In the aged, reduced pulmonary homeostasis, including diminished wound repair and regenerative potential, is attributed to exhaustion of lung stem cell population.^38^ Therefore, microinjuries to the lung epithelium can elicit incomplete wound repair, triggering a fibrotic response. The presence of activate hypoxia signaling in hyperplastic AEC2s surrounding fibroblastic foci^11^ suggests a possible role of hypoxia in proliferation of AEC2s. Indeed, hypoxia induces AEC2 proliferation via increased doxycytidine kinase (DCK) expression in the lung epithelial cells of IPF patients.^10^ Although little is known about the protein kinase function of DCK, it has been recently shown to increase pFAK1 levels,^39^ which strengthens our observation of FAK1 as a central regulator of epithelial hypoxia signaling.

It is important to note that our studies demonstrate that galectin-1 and FAK-1 reciprocally enhance their activities during hypoxic injury, leading to PF. Indeed, galectin-1 increases plasticity of cancer stem-like lung epithelial cells.^25^ This mechanism may have a critical role in maintaining hyperplasic AEC2s surrounding fibroblastic foci via FAK1 activation. Indeed, our results suggest increased expression of galectin-1 transcript in the hyperplastic areas in the lungs of IPF patients. Our data also suggest that galectin-1 inhibition can interfere with FAK1 activation and causes apoptosis in the lung parenchyma, consistent with recent findings in cancer cells.^40,41^ Further, galectin-1 interacts with integrin *β*1 subunit,^42^ which activates FAK1.^43^ Integrin *β*1 subunit expression is often increased on the surface of airway epithelial cells in response to lung injury, including hypoxia, to promote cell proliferation.^44,45^ Galectin-1, once secreted from hyperplastic AEC2s due to elevated hypoxia signaling, can also interact with integrin *β*1 on the surface of neighboring mesenchymal cells in the fibroblastic foci, enhancing their motility.^46^ Thus, it is likely that in a hypoxic environment, increased galectin-1 expression can activate integrin *β*1/FAK1 signaling axis and promote AEC2 proliferation, rendering them hyperplastic as seen around the fibrotic foci.

Several mouse models have used multiple bleomycin injuries in an attempt to mimic a non-resolving fibrotic phenotype.^43^ However, these models require high doses of bleomycin to trigger a fibrotic phenotype. Therefore, we have developed a PF model in which mild injury associated with hypoxia becomes a physiologically relevant injury factor associated with PF. Although bleomycin alone did not cause any detectable fibrosis in the lung, elevated galectin-1 expression may have predisposed the lung to fibrosis following subsequent insults. Indeed, hypoxia, as a secondary physiological insult enhanced the fibrotic phenotype and had significant decline in lung function. However, galectin-1 inhibition towards the end of inflammatory phase of bleomycin injury (day 6) attenuated development of hypoxia-induced PF. Therefore, galectin-1 represents an attractive target to prevent exacerbations in patients with IPF.

Consistent with the progressive decline in lung function observed in IPF patients, we find that mice exposed to hypoxia following bleomycin injury had significant decline in lung function, which was rescued by galectin-1 inhibition. The fibrotic lungs displayed increased resistance of conducting airways and small airways, suggesting impaired gas exchanged in a stiffened lung.^47–49^ Taken together, our observations suggest that hypoxia signaling caused PF in the distal lung, reminiscent of fibrosis observed in IPF.

In conclusion, our study provides a novel mechanistic insight into the profibrotic role of hypoxia in lung epithelial cells via galectin-1 (Figure 8b). Hypoxia-mediated crosstalk between FAK1, galectin-1, and TGF-*β* signaling in PF. Therefore, the possibility exists that other hypoxia-associated signaling pathways such as Notch, Hedgehog, and Wnt signaling may participate in progression of PF.^26,50,51^ While AE-IPF remains a major cause of mortality in IPF patients, patients with previous histories of lung infections and injury may also be prone to developing PF due to sustained hypoxia signaling in the lung. Therefore, we propose galectin-1 as an important mediator of profibrotic events in the lung and a viable therapeutic target in patients suffering from fibrotic lung diseases, including IPF.

## Materials and Methods

### Cell culture and treatments

Majority of the experiments using lung epithelial cells were performed in H441 cells (ATCC, Manassas, VA, USA; no. HTB-174). H441 cells representing morphological features and barrier functions of normal AECs and club cells^52,53^ are also more alveolar epithelial-like than A549 cells, which do not express any of the surfactant markers and contain similar surfactant phospholipids to fibroblasts.^54,55^ H292 (ATCC; no. CRL-1848), A549 (ATCC; no. CCL-185), and LL24 (ATCC; no. CCL-151) were kindly provided by Dr Eric Haura (H Lee Moffitt Cancer Center and Research Institute, Tampa, FL, USA). IMR-90 (ATCC; no.CLCL-186) and LL-97A (ATCC; no. CCL-191) cells were purchased from ATCC. H441 and H292 cells were cultured in RPMI medium (Sigma, St. Louis, MO, USA); A549, LL97A, and LL24 cells were cultured in F12K medium (Invitrogen, Carlsbad, CA, USA); IMR-90 cells were cultured in Eagle’s minimum essential medium (ATCC). Wnt-reporter cell line (3T3 mouse fibroblasts) was purchased from Enzo Life Sciences (no. ENZ-61002-0001, Farmingdale, NY, USA) and grown in DMEM medium (Sigma). All cell culture media were supplemented with 10% fetal bovine serum and 5% mixture of penicillin G, streptomycin, amphotericin B (Invitrogen), and plasmocin (Invivogen, San Diego, CA, USA). NuLi-1 cells (ATCC; no. CRL-4011) were cultured in bronchial epithelial cell growth medium (ATCC; PCS-300-040). All cell lines were cultured at 37 °C in an incubator that maintained 5% CO_2_. TGF-*β*1 (no. 240-B) and Wnt-3a (no. 5036-WN-010) recombinant proteins were obtained from R&D Systems (Minneapolis, MN, USA).

### Transfections and transcriptional reporter assays

Artificial SMAD reporter (SBE4-Luc), a gift from Bert Vogelstein (Addgene, Cambridge, MA, USA; no. 16527)^56^ and *β*-catenin reporter (TOP-FLASH)^57^ were transfected using the polyethylenimine method^58^ with modifications as described previously.^59^ Wnt reporter fibroblast 3T3 mouse fibroblast cell line was used to assess Wnt- and TGF-*β*-mediated *β*-catenin transcriptional activity with and without FAK-inhibitor. PF-573 228 was used as a FAK inhibitor in the transcriptional studies (Selleckchem, Houston, TX, USA; no. S2013).^60^ The light units were assayed by luminometry (Synergy H4; BioTek, Winooski, VT, USA).

### Isolation and culture of AEC2s

AEC2s were isolated from normal mice using previously described method^61^ with the following modifications: lungs were subjected to dispase I treatment and cells were passed through 100 and 40 *μ*m filters. Red blood cells were lysed using red blood cells lysis buffer (Sigma–Aldrich, St. Louis, MO, USA; no. R7757). AEC2s were grown on fibronectin-coated (10 *μ*g/ml) plates and cultured in DMEM media (ATCC; no. 30-2002) supplemented with 10% fetal bovine serum and 5% mixture of penicillin G, streptomycin, amphotericin B (Invitrogen) and plasmocin (Invivogen). Purity of AEC2s was measured by considering E-cadherin-positive cells as epithelial cells and vimentin-positive cells as non-epithelial cells.

### Immunocytochemistry and immunoblot analysis

For immunocytochemistry (ICC), cells were grown on glass coverslips, and exposed to hypoxia for the specified durations. Cells were fixed in 4% paraformaldehyde, blocked, and permeabilized with 0.1% Triton X-100 and 2% bovine serum albumin. The primary antibodies used were as follows: pSMAD3 (Ser423/425; Cell Signaling, Danvers, MA, USA; no. C24A9), 1 : 100 dilution, incubated overnight at 4 °C; *α*-SMA (Abcam, Cambridge, UK; no. ab5694), 1 : 200 dilution, incubated overnight at 4 °C; FAK (Santa Cruz, Dallas, TX, USA; no. sc-558) and p-FAK (pY397; Santa Cruz; no. sc-558), 1 : 100 dilution, incubated at room temperature for 1 h; E-cadherin (Cell Signaling; no. 3195P), 1 : 50 dilution, incubated overnight at 4 °C; vimentin (Abcam; no. ab92547), 1 : 200 dilution, incubated overnight at 4 °C. Cells were then washed and incubated in appropriate secondary antibodies mixed with phalloidin (Invitrogen; no. A12381) at 1 : 100 dilution for 1 h at room temperature and mounted with a medium containing DAPI (Vector Laboratories, Burlingame, CA, USA; no. H-1500) at 1 : 5 dilution in 1× TBS. Cells were visualized and imaged using an Olympus IX51 microscope and cellSens software (Olympus Corporation, Tokyo, Japan)). Immunoblotting was performed as described previously.^62^ Primary antibodies used were as follows: pFAK (Y397) (Santa Cruz; no. sc-11765-R) at 1 : 500 dilution; *β*-actin (Sigma-Aldrich; no. A0560) at 1 : 1000; *α*-SMA (Abcam; no. ab5694) at 1 : 1000 dilution; HIF-1*α* (Cayman Chemicals, Ann Arbor, MI, USA; no. 10006421) at 1 : 500 dilution; caspase-3 (Sigma-Aldrich; no. C8487) at 1 : 1000 dilution. Peroxidase-conjugated AffiniPure goat anti-rabbit IgG secondary antibody (Jackson ImmunoResearch Laboratories, West Grove, PA, USA; no. 111-035-045) was used at 1 : 10 000 dilution with incubation at room temperature. ICC and immunoblot experiments were quantified using ImageJ (Bethdesa, MD, USA).

### Stable isotope labeling by amino acids in cell culture proteomics and pathway analysis

H441 cells labeled with either light lysine/arginine or heavy lysine lysine(U-^13^C_6_)/arginine(U-^13^C_6_,^15^N_6_; red) isotope were grown for eight doublings. Cells grown with light isotopes were exposed to normoxia (21% O_2_; 72 h; *n*=3) and cells grown with heavy isotopes were exposed to hypoxia (1% O_2_; 72 h; *n*=3). Total proteins were collected from cell lysates and processed via filter-aided sample preparation as described previously.^63,64^ Trypsin-digested peptides were analyzed on a Q-Exactive Plus with a 50 cm UPLC column using a 2 h gradient (2–40% acetonitrile) on an EASY-nLC 1000 System (Thermo Fisher, Waltham, MA, USA). MS/MS spectra were extracted and raw files were searched against the *Homo sapiens* protein sequence database from the universal protein resource database using Mascot (Matrix Science, Boston, MA, USA). Peptide and protein validations were performed using Scaffold (version 3.00.06; Proteome Software, Portland, OR, USA). A total of 1476 significantly deregulated proteins in at least two replicates were identified (Mann–Whitney Test; *P*<0.05). The mass spectrometry proteomics data have been deposited to the ProteomeXchange Consortium via the PRIDE^65^ partner repository with the data set identifier PXD005187 and 10.6019/PXD005187.

### Pathway analysis and network analysis

Data were analyzed using QIAGEN’s Ingenuity Pathway Analysis (IPA; QIAGEN Redwood City; www.qiagen.com/ingenuity).

#### Upstream analysis

This approach determines the cascade of upstream protein regulators that can provide evidence of the differential regulation of proteins observed in hypoxic proteome.^66^ There was a significant overlap between the deregulated proteins from hypoxic proteome and known targets of TGF-*β* and Wnt3a (*P*<0.05). Further, TGF-*β* and Wnt3a were predicted to be upregulated as inferred by the status of the deregulated hypoxic proteins in our analysis. Both TGF-*β* and Wnt3a were assigned an activation *z*-score>2.00 by IPA, which suggested activation of the upstream regulators.

#### Functional enrichment

Significantly deregulated proteins were analyzed by IPA for their functional enrichment, which gives overrepresented functions of the deregulated proteins when compared with the entire human proteome as a reference data set. An FDR ⩽0.05 (−log *P*-value=1.33) was used to reduce false positives and ensure that all cellular functions are derived in an unbiased manner. A network of hypoxia-deregulated proteins involved in cellular movement was constructed using already known crosstalk between these proteins; this crosstalk was established by IPA via literature mining. FAK1 was identified as one of the proteins that can influence hypoxia-deregulated proteins based on known functional relationships between FAK1 and its effector proteins that are differentially regulated in our hypoxia proteome.

### Hypoxia treatment of cells

Different cell types were incubated in an airtight Plexiglass chamber with a digital oxygen sensor (BioSpherix, Parish, NY, USA). ProOX110 regulator (BioSpherix) was used to maintain oxygen concentration at 1% for the indicated time periods unless otherwise noted. The chamber was kept in a tissue culture incubator to maintain 37 °C temperature and 5% CO_2_.

### Electric cell-substrate impedance sensing (ECIS)

For wound healing assay, H441 or FAK-null H441 cells were grown to confluence on ECIS culture ware (8W1E; Applied Biophysics, Troy, NY, USA). Cells were wounded using an elevated field pulse of 3000 mA at 80 000 Hz applied for 10 s, producing a uniform circular lesion 250 *μ*m in size. The impedance was measured at 16 000 Hz, normalized to its value at the start of data acquisition and plotted as a function of time. For proliferation assay, 10 000 cells were seeded in each well on ECIS cultureware (8W10E+; Applied Biophysics). After allowing the cells to attach, cells were serum starved for 16 h, before introducing media replenished with 10% bovine serum. The resistance was measured at 4000 Hz for up to 160 h.

### RNA isolation and real-time PCR analysis

Total RNA was isolated from cells as described previously.^59^ Five hundred nanograms of purified RNA was converted into cDNA using the qScript cDNA SuperMix (Quanta Biosciences, Beverly, MA, USA; no. 95048-100) and used for real-time polymerase chain reaction (RT-PCR) assays. RT-PCR assays were set up as described previously.^59^ Primer sequences are provided in Supplementary Table S3.

### Generation of FAK-null lung epithelial cells

H441 cells were infected with lentiviral particles expressing shRNA targeting FAK1 transcript. (Sigma-Aldrich; no. TRCN0000121129). Viral infection was performed for 8 h in RPMI-1640 media containing 10 *μ*g/ml of polybrene. Cells were washed with phosphate-buffered saline (PBS) and replenished with normal media. Puromycin selection (2 *μ*g/ml) identified cells with stably integrated shRNA. FAK1 targeting (FAK1-null) was confirmed by immunoblotting for FAK1 expression. FAK1-null H441 cells were maintained in RPMI media containing 2 *μ*g/ml puromycin.

### Cell proliferation and migration assays

Cell proliferation assays were performed using Cell Counting Kit-8 (Fluka; Biochemika, Ronkonkoma, NY, USA) as described previously^62^ with the following modifications: H441 cells were plated in 96-well plates at described quantities and cultured in appropriate growth medium. Cells were serum starved overnight and exposed to either hypoxia or normoxia for indicated time period. Cell migration was measured by scratch assay. H441 cells were grown on 10 cm^2^ cell culture dish to 100% confluence. Scratches were made using sterile pipettes and imaged after changing media (0 h). H441 cells were exposed to normoxia or hypoxia for the indicated time period. At least three scratches were made in each experimental group and 10 images at ×40 were taken of each scratch to be used for quantitation. Images of the scratches were taken every 24 h, unless otherwise indicated. Cell migration was measured as the percent of wound closure compared with control cells using ImageJ.

### Treatment of mice

The 129X1/SvJ mice (no. 000691) were obtained from Jackson Laboratory (Bar Harbor, ME, USA) and housed in humidity- and temperature-controlled rooms on a 12 h light–dark cycle with food and water. Four- to 6-week-old mice were exposed to 50 *μ*l of 0.05 U bleomycin (Selleckchem Chemicals; no. S1214) or saline via intratracheal instillation on day 0. Mice were exposed to hypoxia (13% O_2_, maintained in a chamber A-15274-P; BioSpherix) or normoxia (normal room air) on day 7 and lungs were harvested on day 21 (*n*=4 in each experimental group). Galectin-1 inhibitor OTX008 was used at 2 mg/kg in 25% EtOH/75% saline via intraperitoneal injections once a day starting on day 5. Animal studies were reviewed and approved by the Institutional Animal Care and Use Committee of University of South Florida.

### Immunohistological staining of mouse lungs

Lungs from experimental or control mice were inflation-fixed with 4% paraformaldehyde in 1× PBS overnight at room temperature. The tissue samples were rinsed in 1× PBS, dehydrated, and embedded in paraffin blocks. Five-*μ*m-thick sections were cut, mounted on microscope slides, deparaffinized, and hydrated. Antigens retrieval was performed by microwaving the slides in 0.1% sodium citrate buffer (pH 6.0) for 45 s. Endogenous peroxide activity was blocked by treating sections with 3% H_2_O_2_ at 37 °C for 10 min. H&E or appropriate primary and secondary antibodies were used to stain tissues. DAB (3,3′-diaminobenzidine) was used as a substrate and hematoxylin was used as a counterstain. DAB intensity was quantified using ImageJ using the ‘IHC toolbox’ plugin as described.^67^ Masson’s trichrome staining was performed as per the manufacturer’s protocol with no modifications (Sigma-Aldrich; no. HT15-1KT).

### Lung function tests

Lung function tests were performed using flexiVent system (SCIREQ Technologies Inc., Montreal, QC, Canada) as described previously.^33^ The flexiVent system comprises of three independent units: (1) Base unit, (2) EC network controller that communicates with the computer, and (3) XC accessory controller that controls aeronob nebulizer for aerosol delivery into the mouse lungs. Briefly, mice were sedated using dexmedetomidine at 0.5 mg/kg, intraperitoneally 10 min before the treatment with an anesthetic agent ketamine at 100 mg/kg, intraperitoneally. The mice were kept under isoflurane at 3% throughout the intubation and during lung function tests. The mice were orotracheally intubated with 18 G catheters (BD Insyte, Franklin Lakes, NJ, USA; no. 8366841) and then attached with the flexiVent system. The mice were ventilated at the tidal volume of 10 ml/kg with an average breathing frequency of 150 breaths/min. Respiratory mechanics were measured as per flexiVent system’s standard forced oscillation technique that applies an oscillatory waveform to the airways of mice and measures pressure, flow, and volume signals. Following perturbations were performed to collect different parameters: Snapshot-150 perturbation that measures resistance, elastance, and compliance. Quick Primer-3 perturbation that measures conducting airway resistance and small airway resistance or tissue damping. Pressure–volume loop perturbation measured airspace closure (area) that indicates airway collapse.

### Collagen quantitation

Total collagen levels from sections of FFPE lungs were quantified using Sirius Red/Fast Green Collagen Quantitation Kit (no. 9046) from Chondrex Inc. (Redmond, WA, USA) according to the manufacturer’s protocol. Briefly, one 5 *μ*m FFPE sections from at least four mice in each group were deparaffinized and hydrated, followed by incubation with 100 *μ*l dye solution containing Sirius Red and Fast Green for 30 min at room temperature. The sections were then washed in running water for 5 min followed by eluting the bound Sirius Red and Fast Green dyes in 1 ml of extraction buffer. Absorbance of the eluted dye was measured at 540 and 605 nm in triplicate for each sample. Total collagen was measured using the following formula according to the manufacturer’s protocol:

$$\mathrm{Total}\;\mathrm{collagen}\;\left(µg/\mathrm{section}\right)=\frac{\mathrm{OD540}-(\mathrm{OD650}*0.291)}{0.0378}$$

The collagen values were then normalized to the average values from four mice in control group.

### Proximity ligation assay

PLA Kit was purchased from Sigma-Aldrich (no. DUO92101). H441 cells were plated onto coverslips and fixed with 4% paraformaldehyde followed by blocking with 30 min. Cells were incubated with primary antibodies against FAK1 (Santa Cruz; no. sc-558) and galectin-1 (ThermoFisher Scientific; no. 43-7400) for 2 h at room temperature. Rest of the protocol was as per the manufacturer. Interactions were visualized and imaged using an Olympus IX51 microscope and cellSens software (Olympus Corporation).

### Analysis of microarray data of human samples

We analyzed the microarray data available at the GEO (Gene Expression Omnibus) repository of genomics data hosted by the NCBI (National Center for Biotechnology Information). GEO data set GSE47460 was analyzed to detect galectin-1 transcript levels in normal lungs and lungs of IPF patients. GSE47460 data set reports microarray data from samples from healthy lungs and the lungs of various ILD patients, obtained from Lung Tissue Research Consortium (LTRC; https://ltrcpublic.com/). Samples obtained from patients suffering from ILDs other than IPF were excluded. Selected samples are listed in Supplementary Tables S1 and S2. GEO data set GSE35309 was analyzed to detect the localization of galectin-1 transcript in normal-looking regions or fibrotic foci or hyperplastic regions of the lungs of IPF patients. Normalized microarray data were analyzed using GEO2R after applying log 2 transformation. GEO2R, an interactive web tool, allows for comparison of original submitter-supplied processed data (https://www.ncbi.nlm.nih.gov/geo/geo2r/). Outliers were removed in the samples by performing Tukey’s test.^68,69^
