# Supplementary material for: Galectin-1 inhibition attenuates profibrotic signaling in hypoxia-induced pulmonary fibrosis
Source: Cell Death Discov. 2017 Apr 10;3:17010–. doi: 10.1038/cddiscovery.2017.10 (PMC5385413; doi:10.1038/cddiscovery.2017.10)
Supplement: Supplementary Information [file cddiscovery201710-s1.docx]

**Supplementary Figure S1: Confirmation of Primary murine AEC2s**

Primary murine AEC2s were plated on coverslips coated with fibronectin and stained with either E-Cadherin (AEC2 marker) or Vimentin (mesenchymal cell marker) with DAPI as nuclear staining. Less than 10% contamination of Vimentin^+^ cells were observed (a total of 3 coverslips with 5 fields/coverslips were analyzed). Scale bar = 100 µm.

**Supplementary Figure S2: Wnt/β-Catenin pathway was activated in hypoxic lung epithelial cells.**

**(a)** Synergistic increase in β-catenin reporter activity following treatments with TGF-β and CoCl_2_ together. Transfection of the β-catenin reporter (TOP-Flash) and its mutant control reporter (FOP-Flash) was performed in H292 lung epithelial cells, which were then treated with either TGF-β (5ng/mL) or CoCl­_2_ (100 µM) or both. p < 0.05 (student’s t-test). Data are shown as mean ± SE (n=3 independent experiments). **(b)** Wnt3a treatment (20ng/mL; 15 minutes) increased pFAK1 (Y397) levels. p < 0.05 (student’s t-test). Data are shown as mean ± SE (n=3 independent experiments).

**Supplementary Figure S3: Generation of FAK-null H441 cells.**

H441 cells were transduced with 4 lentiviral constructs harboring shRNA targeting FAK1 and stable shRNA expressing lines were selected using puromycin (1µg/mL). Protein lysates obtained from these stable cell lines were screened for FAK1 expression by immunoblotting. Cell line with the least expression of FAK1 (lane 2) was selected for further experiments and are referred to as “FAK-null cells” for brevity.

**Supplementary Figure S4: Galectin-1 inhibition attenuated hypoxia-induced FAK1 activation and activated Caspase-3 *in vitro* and *in vivo*.**

**(a)** Hypoxia induced pFAK1 (Y397) expression and reduced Active Caspase-3 expression in the lungs of mice injured with subclinical levels of bleomycin. Galectin-1 inhibition (OTX008) reduced pFAK1 expression and increased Caspase-3 activation in the lungs of mice injured with bleomycin followed by exposure to hypoxia. Levels of pFAK1 and Caspase-3 were analyzed using ImageJ software and presented as arbitrary units (A.U.). *p < 0.05 (One-Way ANOVA followed by post-hoc test). n.s. = not significant. Data are shown at average of at least 6 images per lobe of the lung ± SE. n=3 lobes were analyzed. Scale bar = 40 µm. **(b)** Hypoxia (1% O_2_; 24 hours) significantly increase pFAK1 (Y397) expression and reduced active Caspase-3 expression. Galectin-1 inhibition (OTX008; 5µM for 24 hours) significantly reduced pFAK1 (Y397) and increased active Caspase-3 expression in hypoxic H441 cells. p < 0.05 (student’s t-test). Data are shown as mean ± SE (n=3 independent experiments).

**Supplementary Figure S5: Galectin-1 expression was higher in hyperplastic regions of lungs of IPF patients**

Significantly higher Galectin-1 transcript levels were observed in hyperplastic regions of the lung of IPF patients (GEO dataset GSE35309). The normalized Galectin-1 transcript levels were compared after removing outliers (see methods). p < 0.05 (One-Way ANOVA followed by post-hoc test).

**Supplementary Tables**

**Supplementary Table S1: Demographic and Functional Characteristics of Healthy Controls**

**Supplementary Table S2: Demographic and Functional Characteristics of IPF Patients**

**Supplementary Table S3: Primer Sequences**
