# Supplementary figures and images for: Galectin-1 inhibition attenuates profibrotic signaling in hypoxia-induced pulmonary fibrosis
Source: Cell Death Discov. 2017 Apr 10;3:17010–. doi: 10.1038/cddiscovery.2017.10 (PMC5385413; doi:10.1038/cddiscovery.2017.10)

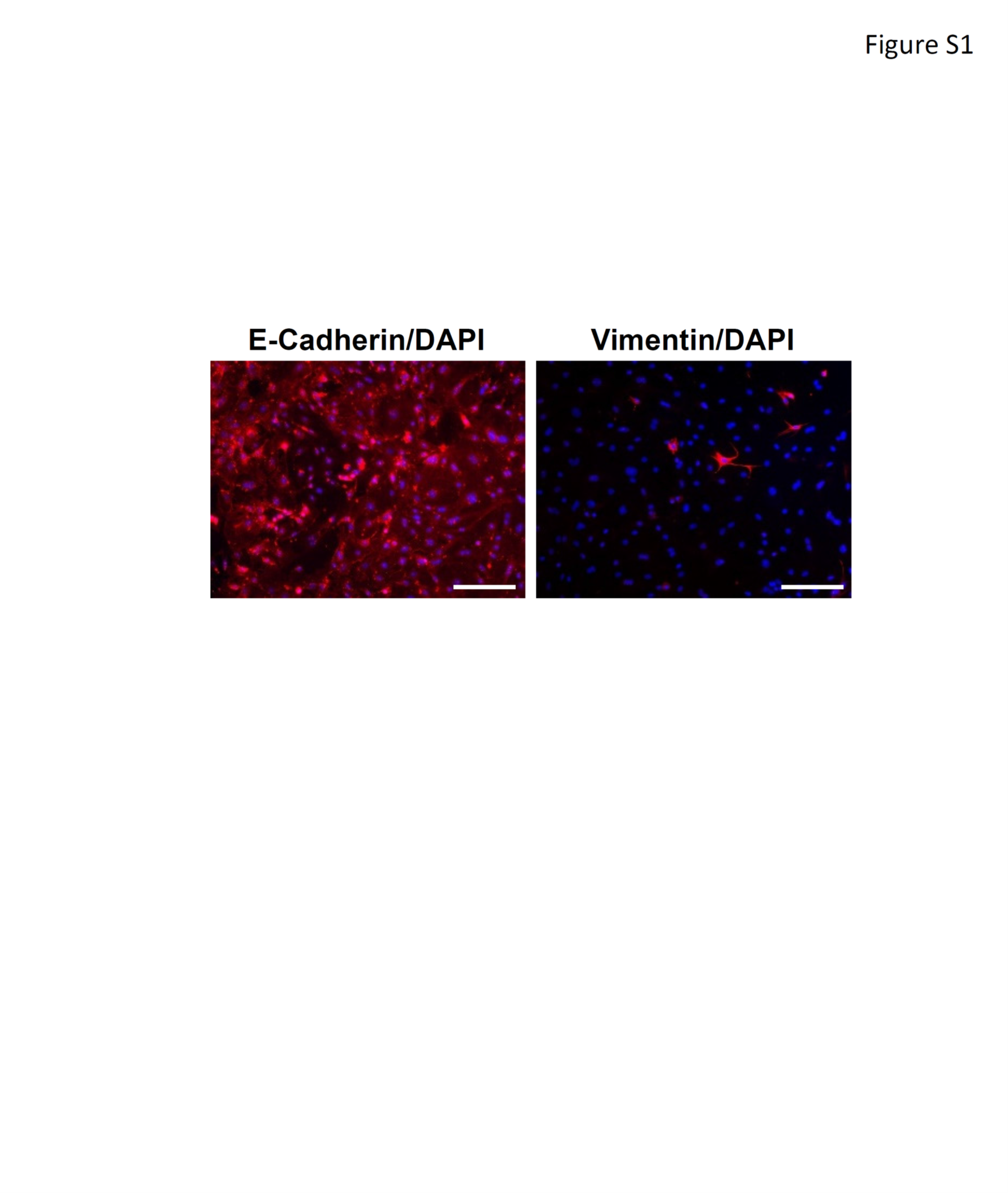

Supplement: Supplementary Figure S1 [file cddiscovery201710-s2.tiff]

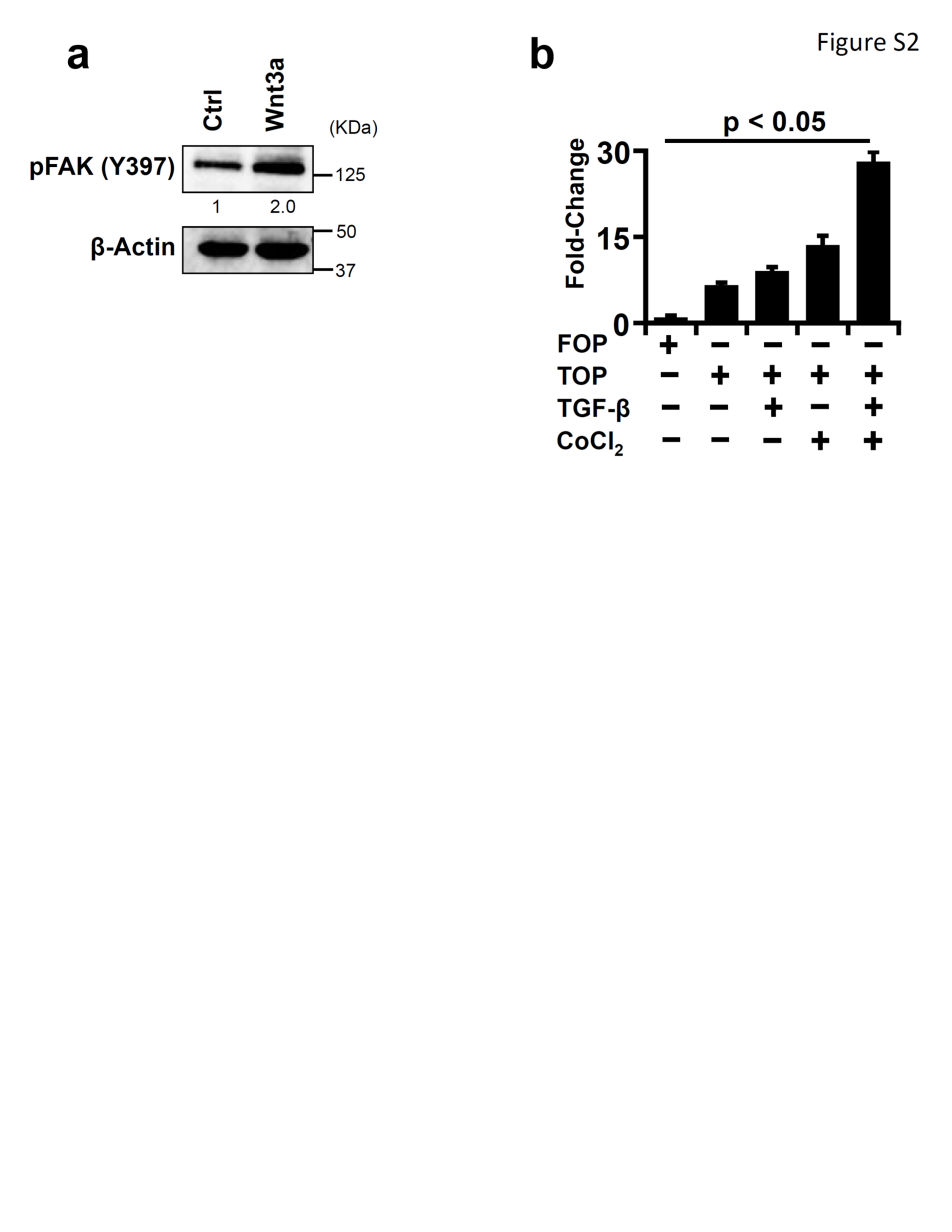

Supplement: Supplementary Figure S2 [file cddiscovery201710-s3.tiff]

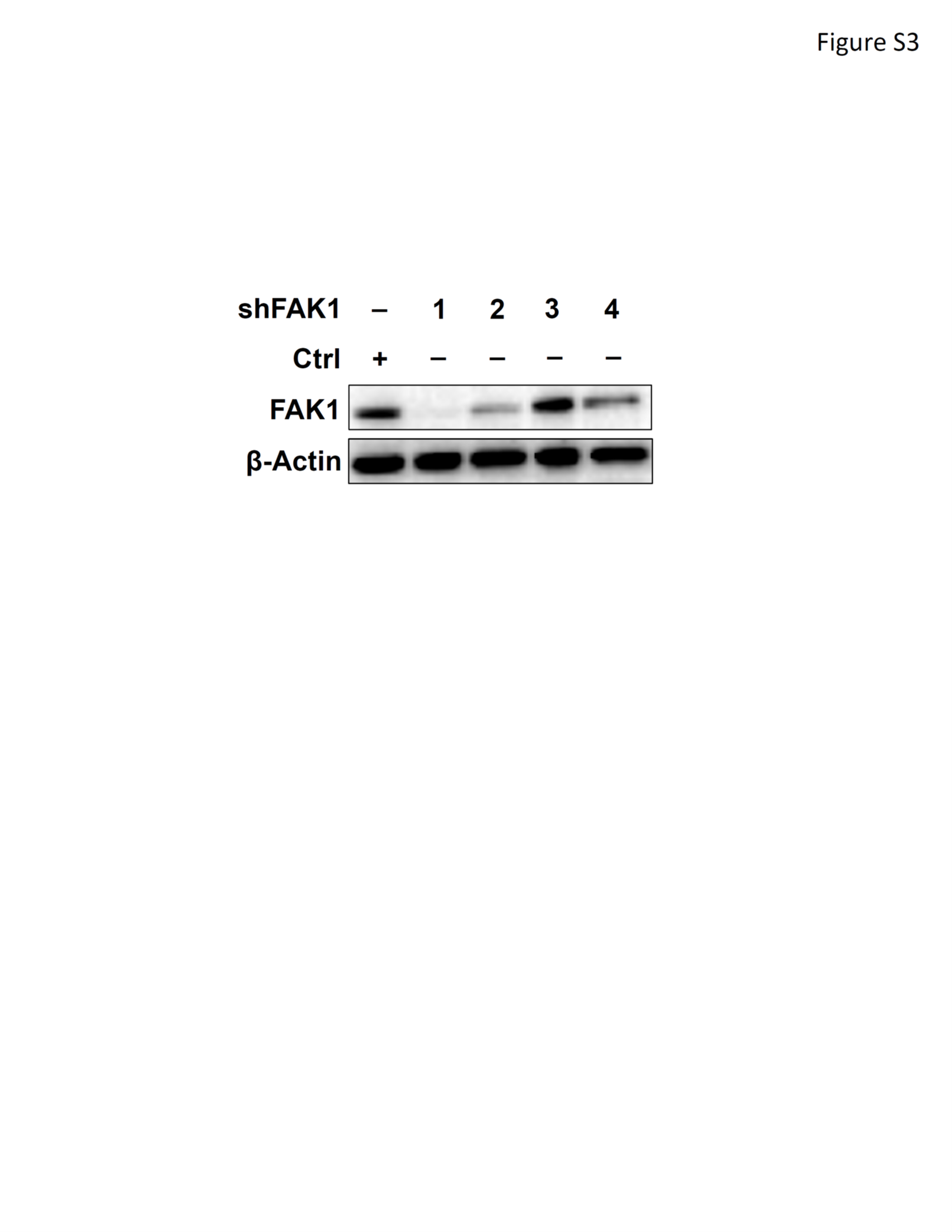

Supplement: Supplementary Figure S3 [file cddiscovery201710-s4.tiff]

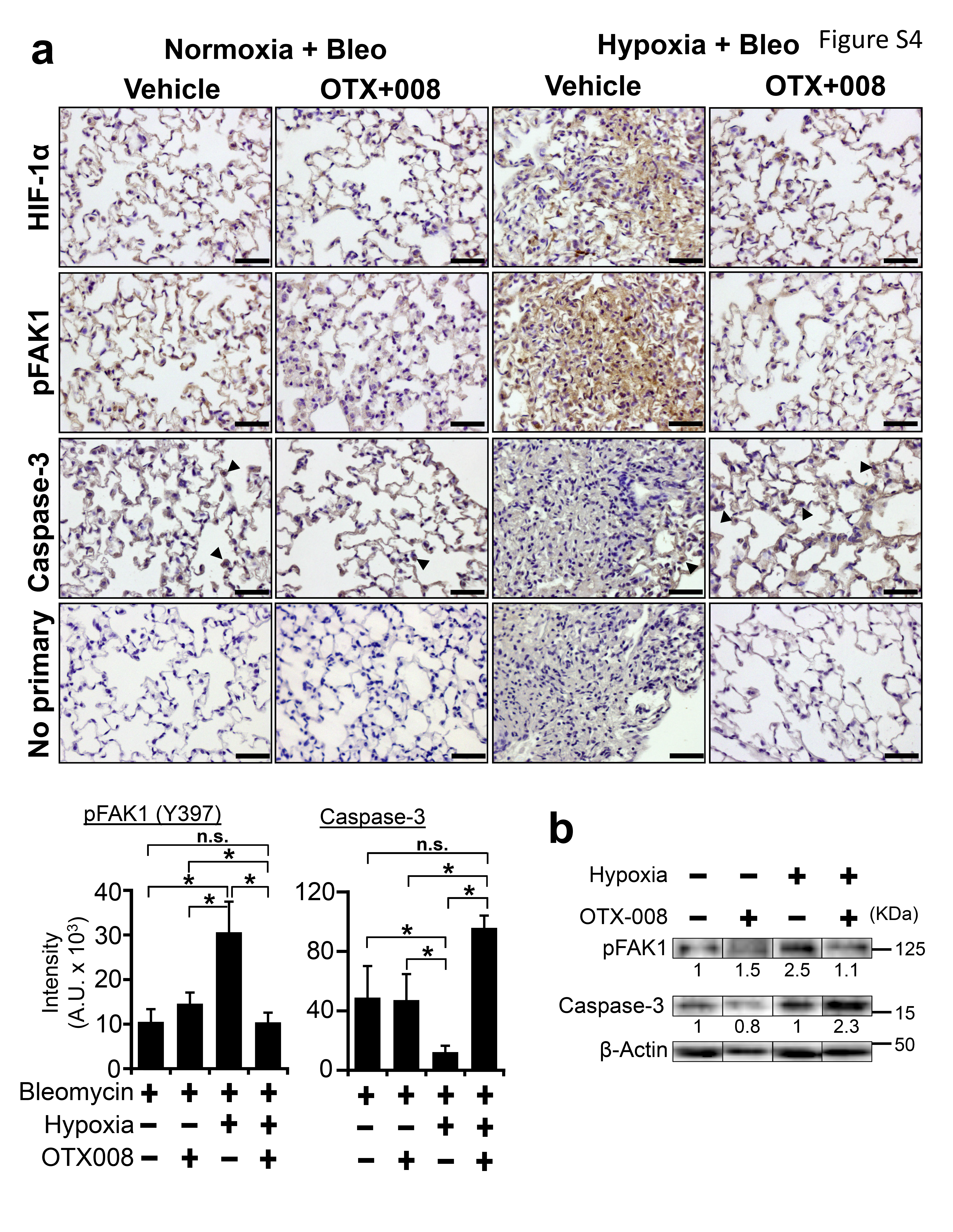

Supplement: Supplementary Figure S4 [file cddiscovery201710-s5.tiff]

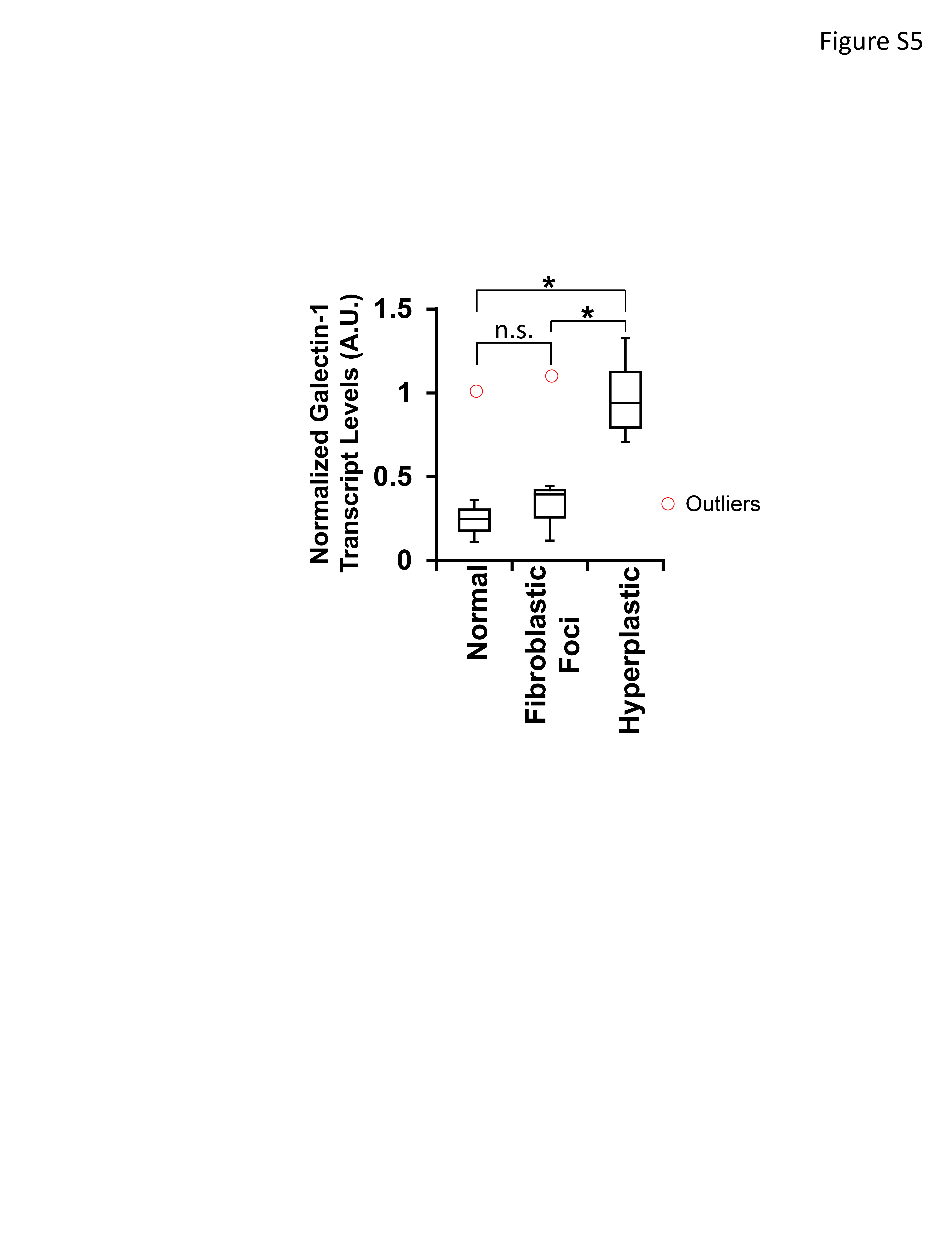

Supplement: Supplementary Figure S5 [file cddiscovery201710-s6.tiff]
